# Clinical Impact of Artificial Intelligence-Based Triage Systems in Emergency Departments: A Systematic Review

**DOI:** 10.7759/cureus.85667

**Published:** 2025-06-09

**Authors:** Abubaker Zakria Ahmed Abdalhalim, Sheimaa Nasreldein Nureldaim Ahmed, Ahmed Mohamed Dawoud Ezzelarab, Mawada Mustafa, Mohammed Gabralla Ali Albasheer, Roaa Eltag Abdelgadir Ahmed, Mowafag Bushra Galal Eldin Elsayed

**Affiliations:** 1 Emergency Medicine, Rustaq Hospital, Rustaq, OMN; 2 General Medicine, Sheikh Khalifa General Hospital, Ummalquwain, ARE; 3 General Practice, Saudi Airline Medical Services Company, Jeddah, SAU; 4 Surgery, Faculty of Medicine, Jazan University, Jazan, SAU; 5 Emergency Medicine, Barka Polyclinic, Barka, OMN; 6 Emergency Medicine, King Fahad Hospital, Albahah, SAU; 7 Emergency Medicine, Mayo University Hospital, Castlebar, IRL

**Keywords:** artificial intelligence, clinical decision support, emergency medicine, machine learning, triage

## Abstract

Emergency departments (EDs) worldwide face increasing pressure to optimize triage processes amidst rising patient volumes and resource constraints. Artificial intelligence (AI) has emerged as a potential solution to enhance triage accuracy and efficiency, yet its real-world clinical impact remains inadequately characterized.

We conducted a systematic review following Preferred Reporting Items for Systematic Reviews and Meta-Analyses (PRISMA) 2020 guidelines, searching PubMed/Medical Literature Analysis and Retrieval System Online (MEDLINE), Excerpta Medica Database (Embase), Web of Science, and Institute of Electrical and Electronics Engineers (IEEE) Xplore (2020-2025) for studies evaluating AI-based ED triage systems. From 119 initially identified records, six studies met inclusion criteria after duplicate removal (n=67), title/abstract screening (n=52), and full-text assessment (n=12). Eligible studies reported quantitative outcomes on AI performance compared to traditional triage methods. Risk of bias was assessed using an adapted Quality Assessment of Diagnostic Accuracy Studies-2 (QUADAS-2) tool. Narrative synthesis was employed due to methodological heterogeneity.

The included studies (n=6) demonstrated AI’s potential to reduce triage time, improve documentation accuracy, and enhance decision support. Voice-based artificial intelligence (Voice-AI) systems achieved 19% faster documentation versus manual methods, while machine learning algorithms reduced mis-triage rates by 0.3-8.9%. However, limitations included undertriage risks, variable accuracy, and predominance of single-center studies. Implementation challenges encompassed workflow integration barriers and insufficient clinician acceptance metrics. AI-based triage systems show promise for improving ED efficiency but require rigorous multi-center validation and standardized outcome reporting. Key gaps include evidence on patient-centered outcomes, equity considerations, and long-term impact studies. Future development should prioritize explainable algorithms, clinician engagement, and ethical frameworks to ensure safe implementation.

## Introduction and background

The integration of artificial intelligence (AI) into healthcare systems represents one of the most transformative developments in modern medicine, with emergency departments (EDs) standing to benefit significantly from these technological advancements [1]. EDs worldwide face mounting pressures from increasing patient volumes, overcrowding, and resource constraints, all of which compromise the timely delivery of care and contribute to adverse clinical outcomes [2]. Traditional triage systems, while foundational to emergency medicine, often rely on subjective clinician judgment and standardized protocols that may not fully account for the complexity and variability of patient presentations [3]. These limitations have spurred growing interest in AI-based triage systems, which promise to enhance decision-making accuracy, reduce waiting times, and optimize resource allocation through data-driven approaches [4]. However, despite the proliferation of AI applications in healthcare, the clinical impact of these systems in real-world ED settings remains inadequately characterized, with existing studies often limited by small sample sizes, methodological heterogeneity, and a lack of standardized outcome measures.

In emergency settings, AI technologies function by integrating machine learning (ML), natural language processing (NLP), and real-time data analysis to support triage decisions. For example, NLP tools can transcribe and interpret patient narratives during triage, while ML models analyze patterns in vital signs and presenting symptoms to predict urgency levels or likely diagnoses [2]. Accurate triage is clinically critical: over-triaging can lead to unnecessary resource utilization and ED crowding, while under-triaging risks delayed care for patients with time-sensitive conditions such as sepsis, stroke, or myocardial infarction [3].

The potential of AI to revolutionize emergency triage lies in its ability to process vast amounts of multimodal data (including clinical histories, vital signs, imaging, and even natural language) to generate actionable insights in real time [5]. Machine learning algorithms, for instance, can identify subtle patterns in patient data that may elude human clinicians, enabling earlier detection of high-risk conditions such as sepsis, stroke, or acute coronary syndromes [6]. Similarly, natural language processing (NLP) systems can streamline documentation processes, reducing administrative burdens and allowing healthcare providers to focus on direct patient care. Yet, for all their promise, AI-based triage systems also introduce new challenges, including concerns about algorithmic bias, model interpretability, and the risk of over-reliance on automated decision-making in high-stakes clinical environments [7]. The ethical implications of deploying AI in emergency settings are equally pressing, as errors or delays in triage can have life-threatening consequences, raising questions about accountability, transparency, and equity in AI-driven healthcare [8].

Despite these challenges, the literature on AI-based triage systems has expanded rapidly in recent years, with studies reporting mixed results regarding their efficacy, safety, and usability. Some investigations have demonstrated significant improvements in triage accuracy and efficiency, while others highlight persistent gaps in validation and implementation [9]. This variability underscores the need for a comprehensive synthesis of the available evidence to clarify the clinical impact of these technologies and identify key areas for future research. To date, no systematic review has critically appraised the performance of AI-based triage systems across diverse healthcare settings while also addressing the practical and ethical challenges associated with their deployment. This gap in the literature is particularly concerning given the accelerated adoption of AI solutions in clinical practice, often without robust evidence to support their widespread use.

This study seeks to address these gaps by evaluating the clinical impact of AI-based triage systems in EDs, with a focus on their ability to improve patient outcomes, streamline workflows, and reduce healthcare costs. By synthesizing findings from a carefully selected body of recent studies, we aim to provide a nuanced understanding of the strengths and limitations of these technologies, as well as their readiness for real-world implementation. Our analysis will also explore the methodological rigor of existing research, highlighting common pitfalls such as insufficient external validation, lack of clinician input in system design, and inadequate attention to patient-centered outcomes. Ultimately, this review aims to inform clinicians, researchers, and policymakers about the current state of AI in emergency triage, while offering evidence-based recommendations for future development and deployment. In doing so, we hope to contribute to the responsible integration of AI into emergency medicine, ensuring that these technologies are implemented in ways that prioritize patient safety, equity, and clinical utility.

## Review

Methodology

This systematic review was conducted following the Preferred Reporting Items for Systematic Reviews and Meta-Analyses (PRISMA) 2020 guidelines [10] to ensure a rigorous and transparent approach to evidence synthesis. The study protocol was designed to systematically evaluate the clinical impact of AI-based triage systems in EDs, with careful consideration of their implementation features, outcomes, and limitations. The protocol was not registered on PROSPERO because this review was conducted as part of an academic assignment with limited time for protocol registration

Search Strategy and Data Sources

A comprehensive literature search was performed across four major databases: PubMed/MEDLINE, Embase, Web of Science, and IEEE Xplore. These databases were selected to ensure coverage of both clinical and technical literature relevant to AI applications in emergency medicine. The search strategy incorporated a combination of controlled vocabulary terms, such as Medical Subject Headings (MeSH) headings in PubMed, and free-text keywords related to AI, machine learning, emergency triage, and clinical decision support. The search was restricted to studies published in English between January 2020 and December 2025 to focus on recent advancements in the field. The full search strategy, including the specific terms and syntax used for each database, is included in Appendix 1.

Study Selection and Eligibility Criteria

Studies were included if they evaluated an AI-based triage system in an ED setting and reported quantitative or qualitative outcomes related to clinical impact, such as triage accuracy, time efficiency, or patient outcomes. Only original research studies, including randomized controlled trials, prospective or retrospective cohort studies, and cross-sectional studies, were considered. Non-empirical studies, such as reviews, editorials, or conference abstracts without full texts, were excluded, as were studies focusing on non-ED settings or non-AI-based decision support tools. Two independent reviewers screened titles and abstracts, followed by a full-text review of potentially eligible studies. Any discrepancies were resolved through discussion or consultation with a third reviewer to ensure consensus.

Data Extraction and Quality Assessment

A standardized data extraction form was used to collect key information from each included study, such as study design, setting, sample size, type of AI system, comparator, and primary and secondary outcomes. Data on implementation challenges, user acceptance, and real-world applicability were also extracted. The methodological quality and risk of bias of each study were assessed using an adapted the Quality Assessment of Diagnostic Accuracy Studies-2 (QUADAS-2) tool [11], which evaluated domains including patient selection, index test (AI system), reference standard, and flow and timing. Additionally, the applicability of the findings to real-world ED settings was appraised to contextualize the evidence.

Narrative Synthesis of Findings

Given the significant heterogeneity in study designs, AI methodologies, and outcome measures across the included studies, a meta-analysis was deemed inappropriate. Instead, a narrative synthesis was employed to provide a comprehensive and nuanced interpretation of the evidence. This approach was chosen to accommodate the diverse nature of the studies, which varied in their AI models, target outcomes, and evaluation metrics. The narrative synthesis allowed for a detailed exploration of the clinical and methodological diversity, as well as the real-world relevance of the findings. The synthesis was structured around key thematic areas, including the impact of AI on triage accuracy and efficiency, safety and clinical outcomes, implementation barriers and facilitators, and ethical and practical considerations. This method ensured that the complexities and contextual factors of AI integration in EDs were adequately addressed.

Transparency and Reproducibility

To uphold the principles of transparency and reproducibility, all stages of the review process--from the development of the search strategy to data extraction and quality assessment--were meticulously documented. This systematic and documented approach minimized potential biases and enhanced the reliability of the review findings. By adhering to these rigorous methodologies, this review provides a balanced and evidence-based assessment of the current state of AI-based triage systems in emergency medicine.

Results

Selection of Studies

**Figure 1:**
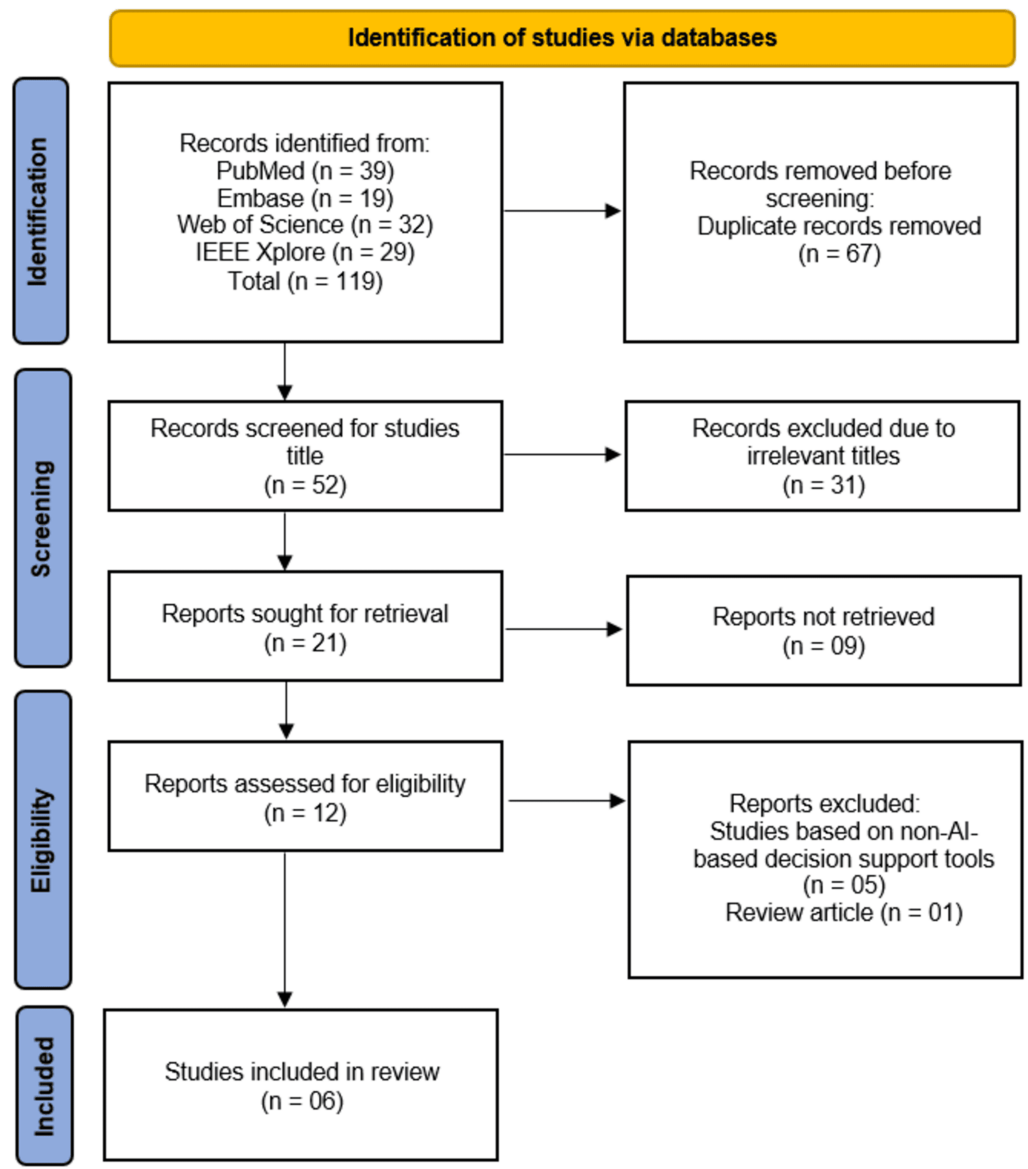
PRISMA Flow Diagram

The systematic search identified 119 records from four databases (PubMed: 39, Embase: 19, Web of Science: 32, IEEE Xplore: 29), with 67 duplicates removed. After screening 52 titles, 31 irrelevant records were excluded. Of the 21 full-text reports sought, 9 were unavailable, leaving 12 reports for eligibility assessment. Five studies were excluded for evaluating non-AI-based tools, and one was excluded as a review article, resulting in six studies included in the final review (Figure 1).

Overview of Included Studies 

The systematic review included six studies published between 2020 and 2025, evaluating the clinical impact of AI-based triage systems in EDs [12-17]. The studies were conducted across diverse geographical regions, including South Korea [12], Germany [13], Greece [14, 15], Taiwan [16], and China [17], reflecting a global interest in AI applications for triage optimization. The study designs varied, encompassing prospective observational studies, cross-sectional evaluations, and system development trials, with sample sizes ranging from 146 [16] to 22,272 [17] patients. The AI systems employed in these studies included voice-based artificial intelligence (voice AI) with natural language processing (NLP), machine learning (ML) classifiers, fuzzy logic, and neural networks, each targeting specific triage functionalities such as documentation support, urgency prediction, and decision-making assistance (Table 1). 

**Table 1:** Characteristics and Summary of Included Studies Abbreviations: AI, artificial intelligence; NLP, natural language processing; RMIS-AI, real-time medical input system with voice artificial intelligence; PMH, past medical history; MTS, Manchester Triage System; AHS, avoidable hazardous situations; ESI, Emergency Severity Index; ICU, intensive care unit; i-TRIAGE, intelligent triage system; TTAS, Taiwan Triage and Acuity Scale; AUROC, area under the receiver operating characteristic curve; AUC, area under the curve; MLS, machine learning system

Author (Year)	Country	Study Design	Sample Size	Type of AI System Used	Comparator	Outcomes Measured	Key Findings
Cho et al., [12] (2022)	South Korea	Prospective observational study	1063 triage tasks by 19 nurses	RMIS-AI, NLP, speech-to-text	Manual input method	Triage task time, record completion rate, data accuracy	RMIS-AI significantly reduced triage time vs manual method; better at capturing chief concerns/PMH; lower accuracy in categorical variables
Cotte et al., [13] (2022)	Germany	Single-center, prospective, cross-sectional, observational study	378	Symptom assessment app (Ada; Ada Health, Berlin, Germany)	Manchester Triage System (MTS)	Safety of urgency advice, undertriage rate, potential avoidable hazardous situations (AHS)	94.7% triage agreement with MTS; 8.9% undertriage; 5.3% potential AHS; 43.4% of patients not emergency cases—could reduce ED burden
Karlafti et al., [14] (2023)	Greece	Prospective observational study (development of an AI classifier)	332 patients	Neural network (AI classifier)	Not reported	ESI prediction accuracy, days of hospital stay, ICU admission rate, waiting time	AI model predicted ESI with 72.2% F1-score; showed potential to support triage decision-making, though accuracy not yet fully satisfactory
Kipourgos et al., [15] (2022)	Greece	System development and evaluation study	616	MLS, fuzzy logic (i-TRIAGE system)	Not reported	Triage decision accuracy, system success rate	High success rates, particularly with fuzzy logic; potential tool for nurse decision support
Leung et al., [16] (2021)	Taiwan	Prospective observational study	146	Interpretable machine learning model (custom triage prediction system)	Traditional TTAS triage (implicit comparator)	AUROC, accuracy (prediction of hospitalization)	Achieved mean AUROC of 0.836 and mean accuracy of 0.805 in predicting hospitalization
Liu et al., [17] (2021)	China	Retrospective and prospective cohort	22,272	MLS	Traditional triage system	AUC, mis-triage rate	MLS achieved higher AUC (0.875 vs. 0.843) and reduced mis-triage rate (0.9% vs. 1.2%) compared to traditional system.

Clinical Impact of AI-Based Triage Systems 

The integration of AI into ED triage workflows demonstrated measurable improvements in efficiency, accuracy, and resource allocation. Cho et al. [12] reported that a real-time medical input system with voice artificial intelligence (RMIS-AI) significantly reduced triage task time and enhanced documentation of chief concerns and past medical history compared to manual methods, though challenges persisted in categorical variable accuracy. Similarly, Liu et al. [17] observed a reduction in mis-triage rates (from 1.2% to 0.9%) and a higher AUC (0.875 vs. 0.843) with their machine learning system (MLS), underscoring its potential to identify critically ill patients more reliably than traditional systems. 

Safety and agreement with established triage protocols were highlighted in several studies. Cotte et al. [13] found that a symptom assessment app (Ada; Ada Health, Berlin, Germany) achieved 94.7% agreement with the Manchester Triage System (MTS), though an 8.9% undertriage rate and 5.3% potential for AHS indicated room for refinement. Karlafti et al. [14] developed a neural network classifier for ESI prediction, achieving a 72.2% F1-score, which, while promising, underscored the need for further validation to address data imbalance and improve accuracy. 

Risk of Bias Results

The risk of bias assessment revealed variability across studies. Cotte et al. [13], Karlafti et al. [14], and Liu et al. [17] demonstrated low risk across all domains, reflecting robust designs and clear reference standards. Cho et al. [12] exhibited moderate risk due to unclear AI calibration, while Leung et al. [16] faced moderate risk from a small sample size and single-center data. In contrast, Kipourgos et al. [15] exhibited high risk due to prototype-stage limitations, lack of real-world validation, and unclear comparators. These findings highlight the need for cautious interpretation of studies with higher bias risk, particularly those in early development phases (Table 2).

**Table 2:** Risk of Bias Results Using QUADAS-2 Tool

Study (Author, Year)	Patient Selection	Index Test (AI System)	Reference Standard	Flow & Timing	Overall Risk of Bias	Applicability Concerns
Cho et al., [12] (2022)	Low	Unclear	Low	Low	Moderate	Low
Cotte et al., [13] (2022)	Low	Low	Low	Low	Low	Low
Karlafti et al., [14] (2023)	Low	Low	Low	Low	Low	Low
Kipourgos et al., [15] (2022)	Unclear	High	Unclear	High	High	High
Leung et al., [16] (2021)	High	Moderate	Low	Moderate	Moderate	Moderate
Liu et al., [17] (2021)	Low	Low	Low	Low	Low	Low

Implementation Features and Challenges

The implementation of AI-based triage systems varied in maturity, ranging from pilot phases to prospective clinical testing (Table 3). Systems like RMIS-AI [12] and MLS [17] were deployed in real-time clinical settings, demonstrating feasibility and immediate benefits. In contrast, other systems, such as the fuzzy logic-based Intelligent Triage System (i-TRIAGE) [15] and the interpretable ML model by Leung et al. [16], remained in developmental or early evaluation stages, with limited external validation or scalability. 

**Table 3:** Clinical Impact and Implementation Features of AI-Based Triage Systems Abbreviations: AI, artificial intelligence; ED, emergency department; MTS, Manchester Triage System; AHS, avoidable hazardous situations; PMH, past medical history; ESI, Emergency Severity Index; F1, F1 Score (harmonic mean of precision and recall); ML, machine learning; EPs, emergency physicians; AUROC, area under the receiver operating characteristic curve; AUC, area under the curve

Author (Year)	AI Integration Level	Clinical Setting (ED Type)	Triage Functionality	Outcome Improvements Reported	Integration Challenges	User Acceptance	Real-Time Use	External Validation	Implementation Stage
Cho et al., [12] (2022)	Medium (voice AI integrated into triage workflow)	General ED	Voice-to-text triage documentation and support	Reduced triage time; improved documentation of chief concerns and past medical history	Lower accuracy for categorical variables; needs technical improvements	Not directly assessed, but participation by 19 nurses implies acceptance	Yes	No	Pilot implementation (prospective study in real-time clinical setting)
Cotte et al., [13] (2022)	Moderate (app-based, pre-hospital triage support)	Interdisciplinary ED	Patient self-triage with urgency advice	94.7% safety compared to MTS; potential to offload non-emergency cases from ED	Undertriage in 5.3% of cases (potential AHS); further validation needed	Not reported	No	No	Pilot/Research Evaluation
Karlafti et al., [14] (2023)	AI model for automatic prediction (neural network classifier)	ED	Predict Emergency Severity Index (ESI) automatically	Accuracy of classifier: 72.2% F1 score; potential support for decision-making; waiting time reported but no direct improvement claims	Accuracy not satisfactory; data imbalance; need for more data collection	Not reported	Not reported	Not reported	Model creation and initial implementation (pilot phase)
Kipourgos et al., [15] (2022)	Prototype-level (fuzzy logic & ML tested)	General ED	Support triage decisions; suggest specialist; educational scenarios	High accuracy in fuzzy logic; improved decision support	No specialty-trained EPs; system still in test phase	Potentially high (user-friendly aim)	Not reported	Not reported	Development & Testing Phase
Leung et al., [16] (2021)	Moderate	National Taiwan University Hospital, Taiwan	Predicting hospitalization using vitals and chief complaints	AUROC = 0.836; accuracy = 0.805	Relies on small sample size; prospective data collection only in one hospital	Not reported	No	No	Prototype/Early Evaluation
Liu et al., [17] (2021)	Moderate	Single-center ED	Assist in identifying critically ill patients; explainable AI	Reduced mis-triage rate from 1.2% to 0.9%; AUC of 0.875 (vs. 0.843)	Not reported	Not reported	Yes	Internal only	Pilot-tested prospectively

User acceptance and integration challenges were inconsistently reported across studies. While Cho et al. [12] implied nurse participation as a proxy for acceptance, others, like Cotte et al. [13], did not explicitly assess user feedback. Technical limitations, such as the need for improved accuracy in categorical variables [12] or larger datasets to mitigate data imbalance [14], were recurring themes. Additionally, the reliance on single-center data in studies like Leung et al. [16] and Liu et al. [17] highlighted the need for multi-center validation to ensure generalizability. 

Discussion

The findings of this systematic review highlight the growing role of AI in transforming ED triage systems, while also underscoring the challenges that must be addressed before widespread clinical adoption can be achieved. Across the six included studies, AI demonstrated measurable improvements in triage efficiency, accuracy, and decision-making support. For instance, Cho et al. [12] reported that a voice-enabled AI system significantly reduced documentation time and improved the capture of patient histories compared to manual methods, aligning with prior research on NLP applications in healthcare [18]. Similarly, Liu et al. [17] found that their MLS outperformed traditional triage in reducing misclassification rates, reinforcing evidence from earlier studies that AI can enhance diagnostic precision in high-acuity settings [19]. However, the variability in system performance--such as the 72.2% F1-score for ESI prediction in Karlafti et al. [14]--suggests that AI models still require refinement to match the reliability of human clinicians, a concern echoed in recent reviews on AI in emergency medicine [20]. 

A critical observation from this review is the trade-off between innovation and validation. While AI systems like the fuzzy logic-based i-TRIAGE [15] showed promise in controlled environments, their real-world applicability remains uncertain due to limited external validation and small-scale testing. This mirrors broader critiques of AI in healthcare, where pilot studies often fail to address scalability or interoperability with existing workflows [21]. The undertriage rates reported by Cotte et al. [13] (8.9%) further emphasize the risks of premature deployment, as even moderately inaccurate systems could delay care for critically ill patients. These findings align with warnings from the Agency for Healthcare Research and Quality (AHRQ), which has cautioned against overreliance on AI without rigorous safety checks [22]. Notably, the lack of multi-center validation in studies like Leung et al. [16] and Karlafti et al. [14] limits generalizability, a recurring issue in AI research that has been criticized in systematic reviews by Liu et al. [17].

The integration challenges identified in this review--such as user acceptance and technical limitations--reflect broader barriers to digital health adoption. For example, Cho et al. [12] did not formally assess nurse acceptance of their voice-AI system, despite its participatory design, a gap also noted in studies of AI-powered clinical decision support (CDS) tools [23]. This omission is significant because clinician trust is a known determinant of AI adoption [24]. Moreover, the predominance of single-center studies (five of six included papers) raises concerns about selection bias and ecosystem specificity, as ED workflows vary widely across institutions [25]. These limitations underscore the need for standardized evaluation frameworks, such as the Developmental and Exploratory Clinical Investigations of DEcision support systems driven by Artificial Intelligence (DECIDE-AI) guidelines [26], to ensure that future AI triage systems are assessed for both efficacy and real-world usability. 

While artificial intelligence applications have demonstrated considerable potential to enhance triage performance, it is crucial to recognize that such systems require careful methodological scrutiny to ensure validity and real-world applicability. The accuracy and reliability of AI-driven triage tools can be significantly compromised if key design and evaluation principles are overlooked. In this context, Altun et al. [27] provide a critical methodological perspective, outlining frequent pitfalls observed in recent AI-triage studies. These include inadequate blinding procedures, inconsistent triage labeling under ED crowding, limited external validation, and insufficient clinician training in AI-assisted decision-making environments [27]. Incorporating these insights during AI system development and validation phases can help mitigate bias, enhance interpretability, and support reliable benchmarking between human and machine-led decisions. Systematic reviews should thus integrate such methodological dimensions when assessing AI-based triage models to promote evidence-based and ethically sound adoption in clinical practice.

Despite these challenges, the potential of AI to alleviate ED overcrowding--a global crisis exacerbated by the COVID-19 pandemic [28]--cannot be overlooked. Cotte et al. [13] estimated that 43.4% of ED visits in their study were non-urgent, suggesting that AI-assisted triage could redirect low-acuity patients to alternative care pathways. This aligns with economic models predicting that AI could reduce unnecessary ED utilization by 15-20%. However, achieving this requires addressing ethical and regulatory hurdles, such as algorithmic transparency [29] and liability frameworks for AI errors [30]. The explainable AI (XAI) approach used by Leung et al. [16] offers a template for mitigating "black box" concerns, but as this review reveals, most systems still lack interpretability features, a gap that must be prioritized in future development. 

Limitations

This review has several limitations. First, the heterogeneity of AI systems and outcome measures across studies precluded meta-analysis, limiting quantitative synthesis. Second, the focus on recent publications (2020-2025) may have excluded earlier foundational research, though this ensured relevance to current AI capabilities. Third, the predominance of single-center studies and prototype-phase systems [15] may overstate the readiness of AI for real-world deployment. Finally, the lack of patient-centered outcomes (e.g., satisfaction, equity impacts) in the included studies represents a critical evidence gap, as highlighted by the WHO’s guidelines on digital health [31]. 

## Conclusions

While AI-based triage systems demonstrate potential to improve emergency department efficiency and accuracy, significant challenges remain in their real-world implementation and validation. Current evidence reveals promising results in reducing documentation time and improving decision-making, yet limitations in generalizability, clinician acceptance, and standardized evaluation highlight the need for more rigorous, multi-center studies with patient-centered outcomes. For successful integration, future development must prioritize explainable algorithms, workflow compatibility, and equitable performance across diverse populations, ensuring these technologies enhance rather than disrupt emergency care delivery. The path forward requires balanced innovation that combines technological advancement with clinical practicality and ethical considerations to realize AI's full potential in emergency medicine.

## Appendices

Appendix 1

Table 4 presents the search strategy syntax used to conduct the literature search.

**Table 4:** Search Strategy Syntax

Database	Search Terms
PubMed	("Artificial Intelligence"[Mesh] OR "Machine Learning"[Mesh] OR "Deep Learning"[Mesh]) AND ("Emergency Service, Hospital"[Mesh] OR "Triage"[Mesh]) AND ("2020/01/01"[Date - Publication] : "2025/04/31"[Date - Publication])
Embase	('artificial intelligence'/exp OR 'machine learning'/exp) AND ('emergency ward'/exp OR 'triage'/exp) AND (2020-2023)/py
Web of Science	TS=("artificial intelligence" OR "machine learning") AND TS=("emergency department" OR triage) AND PY=(2020-2023)
IEEE Xplore	("All Metadata":artificial intelligence OR "All Metadata":machine learning) AND ("All Metadata":emergency OR "All Metadata":triage) AND Year:2020-2023
